# Adhered macrophages as an additional marker of cardiomyocyte injury in biopsies of patients with dilated cardiomyopathy

**DOI:** 10.1515/med-2024-1099

**Published:** 2025-02-04

**Authors:** Oliwia Warmusz, Arkadiusz Badziński, Edyta Reichman-Warmusz, Damian Dudek, Romuald Wojnicz

**Affiliations:** Department of Histology and Cell Pathology, Faculty of Medical Sciences in Zabrze, Medical University of Silesia in Katowice, Jordana 19, 41-808, Zabrze, Poland; Silesian Nanomicroscopy Center, Silesia LabMed: Research and Implementation Center, Zabrze, Poland; Institute of Linguistics, University of Silesia, Katowice, 40-007, Poland; Department of Surgical Dentistry, University of Nicolaus Copernicus, Jagiellonska 13–15, Bydgoszcz, Poland; Department of Histology and Cell Pathology, Faculty of Medical Sciences in Zabrze, Medical University of Silesia in Katowice, Zabrze, Poland

**Keywords:** heart failure, dilated cardiomyopathy, myocarditis, chronic myocarditis, endomyocardial biopsy, macrophages

## Abstract

**Background:**

Macrophage accumulation found in biopsy specimens of patients with dilated cardiomyopathy (DCM) has been thought to reflect chronic myocarditis. However, it is unsettled whether they are responsible for the active or persistent phase of the disease.

**Objective:**

The aim of this study was to count the number of macrophages in relation to plasma concentrations of cardiac troponin T (cTnT).

**Methods:**

We studied the biopsies of 181 patients with DCM by immunohistochemistry using anti-CD68(+) antibodies. The total number of CD68(+) and the number of CD68(+) cells attached to injured cardiomyocytes were counted and presented as the number of cells/mm^2^.

**Results:**

Two expression patterns of CD68(+) macrophages were observed: those localized freely in the interstitial space only, and the cells attached to injured cardiomyocytes. As regards macrophages adhered to injured cardiomyocytes, 72 out of 181 (39.8%) patients presented these cells in the biopsy sections. Both the total number of CD68(+) macrophages and the number of CD68(+) cells directly adhered correlated negatively with cTnT in the serum of DCM patients (Spearman’s rho, *r* = −0.45, *P* < 0.001 and *r* = −0.31, *P* = 0.009, respectively).

**Conclusion:**

Macrophages attached to injured cardiomyocytes may reflect chronic (ongoing) inflammation in the myocardium.

## 1 Introduction

Dilated cardiomyopathy (DCM) is related to a broad range of progressive disorders characterized by pathological myocardial remodeling in a diffuse or multifocal manner manifested clinically by impaired cardiac contractility [1]. The end stage of the disease leads to symptomatic heart failure (HF) in most patients and is related to high morbidity and mortality rates [2,3].

It has generally been assumed that chronic immune response may play an important role in the development and disease progression [2,4]. Accordingly, we recently reported that a higher number of CD68-positive macrophages in biopsy specimens significantly predicted the clinical progression of HF due to DCM in patients after a 1-year follow-up [5]. These observations confirmed the previously reported impact of macrophages on the prognosis in DCM [6].

Cardiac tissue contains large numbers of resident and infiltrative macrophages, which play a major role in immune response and post-inflammatory tissue remodeling [7,8]. Macrophage family cells have marked phenotypic heterogeneity, which is related to their inflammatory responses [9]. In addition, macrophages are not “permanently differentiated.” They are characterized by plasticity and are able to shift their transcriptional profile depending on the local environment [10,11,12].

So far, all data on macrophages in the myocardium of DCM patients have been based on counting the total number of these cells. However, considering the fact that histological detection of myocarditis (MCI) requires the presence of cell infiltration with myocyte cell necrosis, the detection of CD68(+) macrophages that are in direct contact with injured cardiac myocytes may be another important feature suggesting ongoing inflammation. However, the definition of universal histological criteria for ongoing MCI is still open to debate.

In the context of the above-mentioned cell necrosis, cardiac troponin T (cTnT) is a specific serum marker of myocardial injury, suggesting ongoing myocardial injury [13]. The increased cTnT level may be related to neurohormonal activation, cytotoxic effect of inflammatory cytokines, cell stretch, and the effect of oxidative stress, which may lead to chronic low-level troponin elevation [14]. In addition, it may serve as a marker for HF progression [15]. The prevalence of elevated troponin T is associated with adverse outcomes in chronic HF [16,17].

Considering the above, the aim of this study was to determine the number of CD68(+) macrophages (total number vs those that were in direct contact with injured cardiac myocytes) in biopsy sections in relation to plasma concentrations of cTnT in patients with DCM with a clinical suspicion of MCI.

## 2 Materials and methods

### 2.1 Patients

We studied right ventricular biopsy specimens of 181 consecutive patients with DCM and a clinical suspicion of MCI (159M, 22F). The mean duration of symptoms was 1.8 years. All biopsied patients presented with functional class II and III HF symptoms (New York Heart Association; NYHA) and decreased left ventricular ejection fraction (<40%) in the echo study. Each patient underwent standard clinical assessment, including a detailed history and physical examination, routine blood studies, thyroid function testing, echocardiography, coronary angiography, and an endomyocardial biopsy. cTnT was measured using the EDTA-plasma samples by electrochemiluminescence (Elecsys 2010 analyzer, Roche Diagnostics, Germany). The lowest detection limit for cTnT was <0.01 ng/mL. All patients were on typical therapeutic regimens for HF prior to the biopsy.

### 2.2 Endomyocardial biopsy procedure

Endomyocardial biopsy was performed using a Cordis bioptome (Cordis Corp., Miami, US). The minimum number of five specimens was obtained from the right side of the ventricular septum. The specimens were promptly immersed in sterile saline and transported on ice to the laboratory. All specimens were fixed for 20 min in cold acetone, immersed in embedding medium (OCT compound, Miles Inc, Elkhart, IN), and stored in liquid nitrogen until tested. Cryostat specimens were cut serially into sections of 5 m thickness, air-dried at room temperature, and assayed.

### 2.3 Immunohistochemistry

Immunohistochemistry on cryostat sections was performed using a Leica Bond Max autostainer (Leica Biosystems). The protocol was based on antibody detection, and the samples were stained using the hematoxylin kit (BOND Polymer Refine Red Detection; DS9390; Leica Biosystems). The frozen sections were incubated with murine monoclonal antihuman antibodies anti-CD68(+) macrophages (cloneEBM11) from DAKO, Glostrup, Denmark. The dilution of the antibody was verified in a series of pilot experiments and was 1:200. As a negative control, clone M0PC-21 from Leica Biosystems was used. As a positive control, liver biopsy specimens from patients with chronic active hepatitis were used. All sections from each patient were scanned using the GRUNDIUM Ocus ^®^microscope scanner. The total number of CD68(+) macrophages and the number of CD68 macrophages attached to injured cardiomyocytes were counted and presented as the number of cells per mm^2^. All counts were made using *NIS* Elements software from Nikon.

### 2.4 Statistical analysis

All parametric data were expressed as mean ± SD. Non-normally distributed data were analyzed by non-parametric descriptive statistics and presented as median (1st–3rd quartile). To assess the relationship between quantitative data, the Spearman’s rank coefficient was used. Differences were considered statistically significant when *P* < 0.05. Statistical analyses were performed using PS IMAGO 4.0 (IBM SPSS Statistics 24 software package).


**Ethical approval:** The protocol was approved by the institutional Bioethics Committee.
**Informed consent:** All patients gave their informed consent.

## 3 Results

**Figure 1:**
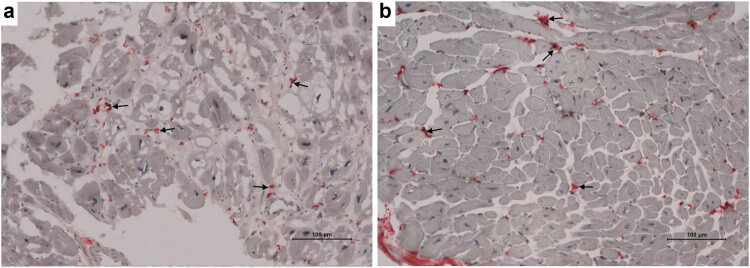
Representative images of CD68(+) macrophages from the frozen samples. (a) Diffusely distributed CD68(+) cells (red color/arrows) localized in the interstitial space (arrows) of the nearly normal myocardium. (b) Another case with free CD68(+) macrophages (red color/arrows).

**Figure 2:**
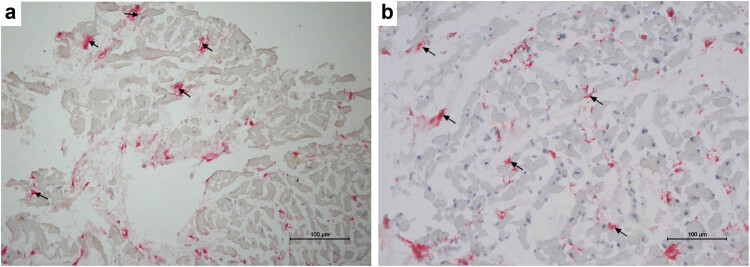
Representative images of CD68(+) macrophages from the frozen samples. (a) CD68(+) macrophages adhered to injured cardiomyocytes (red color/arrows). (b) Another case with activated macrophages (red color/arrows) in tight contact with injured cardiomyocytes with an average of 19.8/mm^2^ cells in this case.

Baseline patient characteristics are given in Table 1. None of the patients presented with hypertension, valvular disease except for relative mitral and/or tricuspid regurgitation, ischemic or systemic heart disease. In this immunohistochemistry study on cryostat sections, we observed two expression patterns of CD68(+) macrophages, i.e., localized freely in the interstitial space (Figure 1a and b) only, and macrophages attached to cardiomyocytes (Figure 2a and b). The mean number of total CD68(+) macrophages and the number of those adhered to injured cardiomyocytes were 26.8 ± 12.9 and 2.67 ± 4.71/mm^2^, respectively. In addition, we revealed an average of total CD68(+) cell number >14 cells/mm^2^ in 100 out of 181 (55.2%) patients. As far as the CD68(+) macrophages adhered to injured cardiac myocytes are concerned, 72 out of 181 (39.8%) samples presented these cells on cryostat sections. Importantly, in 7 positive cases for the presence of adhered macrophages (7 out of 81, 8.64%), the average of total CD68(+) cells was <14 cells/mm^2^.

**Table 1:** Clinical and patient demographic details

**Sex, male/female**	**159/22**
Age, years	41.3 ± 9.9
Male	39.7 ± 7.9
Female	42.4 ± 8.8
Atrial fibrillation, *n* (%)	26 (14.3)
BMI (kg/m^2^)	25.6 ± 3.9
Male	24.8 ± 2.8
Female	26.9 ± 6.1
NYHA, *n* (II/III)	161/20
LVEF (%)	30.1 ± 9.5
LVDD (mm)	65.8 ± 8.8
LVSD (mm)	49.1 ± 8.8
MR (score), median (1st–3rd quartile)	1.2 (1.0–2.2)
C-reactive protein (mg/L), median (1st–3rd quartile)	2.2 (1.2–4.2)
cTnT (ng/mL), median (1st–3rd quartile)	0.005 (0.005–0.10)
NT-proBNP (pmol/L), median (1st–3rd quartile)	497.7 (142.7–1156.5)

Values are expressed as mean ± SD or as indicated. BMI, body mass index; cTnT, cardiac troponin T; MR, mitral regurgitation; NT-proBNP, amino-terminal pro-B-type natriuretic peptide; NYHA, New York Heart Association; LVEF, left ventricular ejection fraction; LVDD, left ventricular diastolic dimension; LVSD, left ventricular systolic dimension.

Based on the immunohistological criteria for MCI published previously [18], only 49 out of 181 (27%) cases presented with MCI in this cohort of patients. Spearman’s rank coefficient showed a significant correlation between the total number of CD68(+) macrophages and CD68(+) cells directly adhered to injured cardiac myocytes (*r* = 0.53, *P* < 0.001). Importantly, both the total number of CD68(+) macrophages and the number of CD68 cells directly adhered correlated negatively with cTnT in the serum of DCM patients (Spearman’s rho, *r* = −0.45, *P* < 0.001, and *r* = −0.31, *P* = 0.009, respectively) (Table 2).

**Table 2:** Association between the total number of CD68(+) macrophages and the number of adhered CD68(+) cells by Spearman’s rank coefficient test

cTnT			
Spearman’s rho	Total CD68(+)	Correlation coefficient	−0.455**
		Sig. (2-tailed)	0.000
		*N*	181
	Adhered CD68(+)	Correlation coefficient	−0.314**
		Sig. (2-tailed)	0.009
		*N*	181

**Correlation is significant at the 0.01 level (2-tailed).

## 4 Discussion

The results of the present study contribute to the available data regarding the use of macrophage infiltration in endomyocardial biopsy as a marker of MCI, especially in the context of chronic (ongoing) form of the disease. Accordingly, this study demonstrated that CD68(+) macrophages could adopt two forms, i.e., the first was characterized by their presence in the interstitial space, without direct contact with the surrounding cardiomyocytes, and the second showed macrophages attached to injured cardiomyocytes using immunohistochemistry. Both of these forms were inversely proportional to cTnT serum levels, which suggested an impaired phagocytic activity of these cells in DCM patients [5].

Currently, there is no doubt that inflammation plays a pivotal role in the pathogenesis of DCM [4]. The longstanding debate on the histological criteria of MCI allowed for setting the quantitative threshold of >14 mononuclear leukocytes per mm^2^ on endomyocardial biopsy samples with the presence of >7 T lymphocytes per mm^2^ for the diagnosis of MCI [18]. However, despite the evolving understanding and diagnostic criteria of myocardial inflammation, universal histological criteria for a chronic form of MCI using endomyocardial biopsy have not been established yet [4,19,20]. Partially, it stems from the fact that histological detection of inflammation requires the presence of a high index of inflammatory cell infiltrations combined with the signs of myocyte injury, which can be hampered by the focal nature of the disease, especially in its chronic form [21]. On the other hand, myocyte injury *per se* is a pathophysiological heterogeneous mechanism, including local ischemia as a major cause [22,23,24].

Based on the immunohistological criteria for MCI published previously [25], only 27% of patients with cut-off values for CD68(+) >14 cells/mm^2^ can be considered positive for having macrophage infiltrations, which indicated MCI in our cohort of patients. These findings are consistent with the results of previous studies by Baccouche et al., where active MCI in endomyocardial biopsy was found in 26% of patients with troponin-I positive acute chest pain in the absence of significant coronary artery disease [26].

Other studies and our previous article recently reported that a higher number of CD68-positive macrophages in biopsy specimens significantly predicted clinical progression of HF due to DCM in patients after a 1-year follow-up [5,6]. Moreover, accumulated clinical and experimental data suggest that chronic inflammation may be responsible for the development and disease progression in at least some of them [4].

## 5 Conclusions

In conclusion, counting CD68(+) macrophages, which are attached to injured cardiomyocytes, may be useful for detecting chronic (ongoing) inflammation in the biopsy specimens of patients with DCM, particularly in those who did not meet the immunohistological criteria for MCI. Further studies are warranted to assess the cut-off value for detecting adhered macrophages that could best reflect chronic inflammation in biopsy sections.
